# Time and labour costs of preventive health care, including vaccinations, in Finnish child health clinics

**DOI:** 10.1371/journal.pone.0270835

**Published:** 2022-10-03

**Authors:** Heta Nieminen, Tuovi Hakulinen, Taneli Puumalainen, Päivi Sirén, Arto A. Palmu

**Affiliations:** 1 Department of Public Health and Welfare, Finnish Institute for Health and Welfare, Tampere, Finland; 2 Department of Public Health and Welfare, Finnish Institute for Health and Welfare, Helsinki, Finland; 3 Department for Safety, Security and Health, Ministry of Social Affairs and Health, Helsinki, Finland; PLOS: Public Library of Science, UNITED KINGDOM

## Abstract

In Finland all children are entitled to regular health check-up visits at child health clinics (CHC). During the visits public health nurses and physicians follow-up the growth and development of the child, evaluate the welfare of the family, give health counselling and vaccinate the children. The aim of this study was to measure the time used by the nurses and physicians for different tasks during the visits and evaluate the costs of preventive health care procedures. Special emphasis was on time and costs used for administering vaccinations. The study was conducted in four CHCs. Trained observers measured the time used for predefined tasks with a stopwatch application operating on a tablet computer. Labour costs of visits and vaccinations were evaluated by using the gross average salary costs of health care personnel. Time used for vaccine logistics and other administrative tasks was obtained by interviewing the nurses in charge of the vaccine logistics at each CHC. Altogether 325 CHC visits of children <13 months were followed. Public health nurse used for a visit in average 49 (range 12–101) minutes, and the corresponding labour costs were 17 (4–35) Euros. Vaccines were administered at 183 visits. Children got on average 2.4 (1–4) vaccine doses per visit. The observed time used for vaccinations was 10.2 (1.6–25) minutes and the costs 3.58 (0.57–8.62) Euros per visit. The observed time included guidance, preparation, administration, and documentation of vaccinations. Adding one dose into a visit increased the time spent on vaccination on average 2.8 minutes (0.99 Euros). The mean non-observed time used for vaccine logistics outside the visits was 3.4 minutes and cost 1.19 Euros per dose. Administering of the vaccines of the Finnish vaccination programme is relatively simple and inexpensive because Finnish children have regular scheduled visits to CHCs.

## Introduction

**Fig 1:**
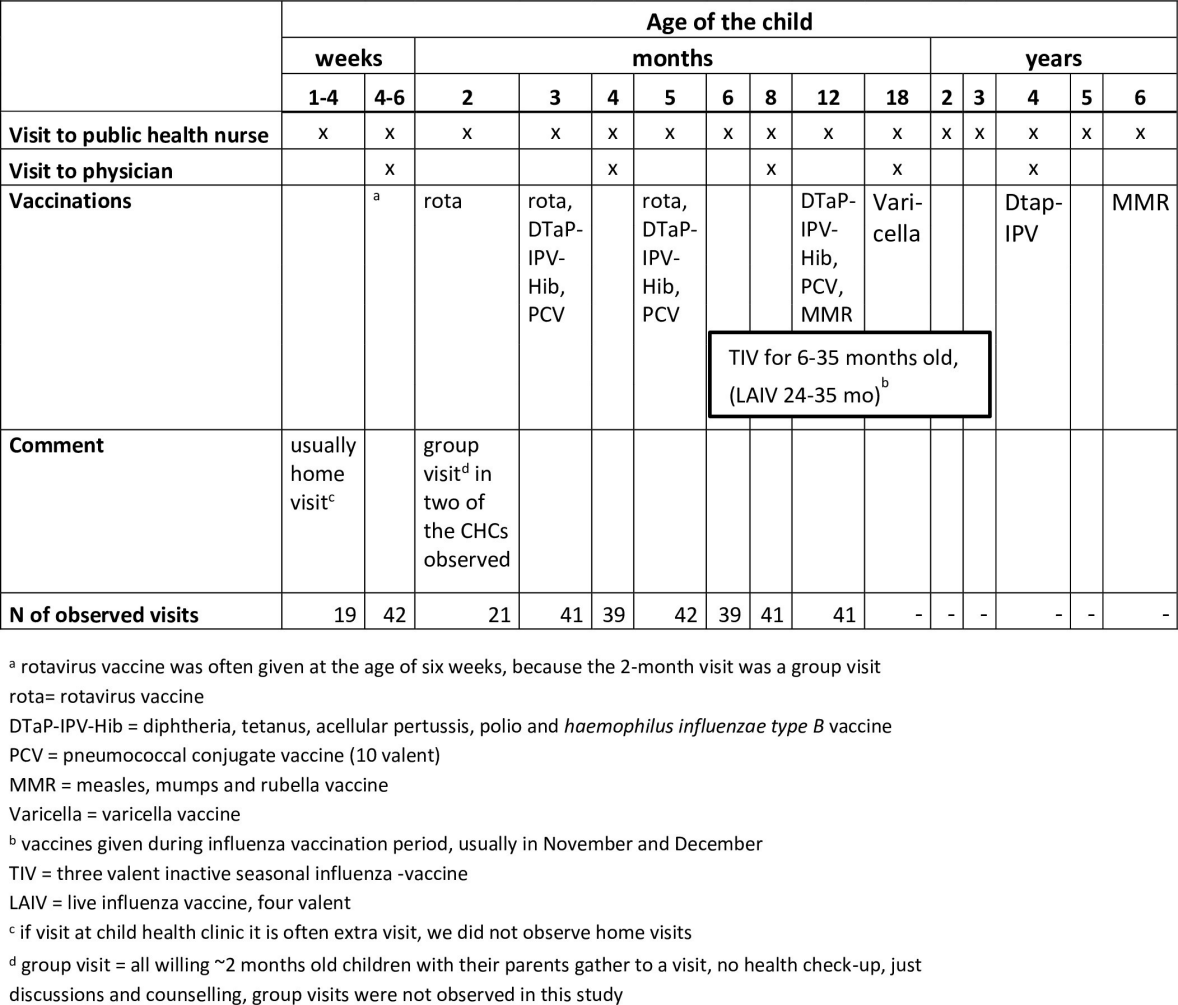
Child-health-clinic visits and vaccination schedule of national vaccination program in autumn 2017.

Child health clinics (CHC) form the backbone of the preventive child health care in Finland. According to law each child should have at least nine regular scheduled health check-up visits during the first year of life and after that at least six health check-up visits between the ages of 1 and 6 (Fig 1) [1]. During the visits physical and psychosocial development and growth of the child are checked. Also the mental health and psychosocial well-being of the family is assessed by questionnaires and interviewing the parent(s). An essential part of the visits is discussion and health counselling of matters concerning health, for example nutrition and sleeping.

The health check-up visits to CHC are mainly conducted by public health nurses (PHN); there are three visits for each child during the first 12 months of life also including also a health check-up, discussions and counselling by a physician (usually general practitioner) [1]. The first PHN visit is often a home visit i.e. the nurse visits the home of the two weeks old child. Another special visit in the schema is a group visit (Fig 1). CHC visits include administering the paediatric vaccines of the national vaccination programme. Vaccines are administered by the PHNs and supervised by physicians.

The overall time which the health care nurse and the physician use for a CHC visit has been previously studied using questionnaires [2] but actual field surveys have not been performed in Finland. By studying the time used by health care personnel (HCP) for different tasks the workflow of the visits can be better understood and it enables the estimation of the actual costs of the various components of the visit. The best way to obtain this kind of data is the time and motion (T&M) method. In the T&M method the action in interest, in this case the CHC visit, is first divided into tasks and after that specially trained observers measure the time used for each predefined task with a stopwatch [3]. The method has been developed for understanding and leaning of industrial work, but as the interest towards effectiveness and efficiency of health care processes has increased the method has been imported from industrial premises to hospitals and health clinics [3]. However, the method is also suitable for assessing the time and costs used for predetermined, fixed tasks without aiming to increase the efficiency of the employees. Previously the T&M method has been used in evaluating the time saved by using combination vaccines [4], fully liquid vaccines [5] or barcode scanning technology for documentation [6].

The costs of vaccination (and vaccine) are needed for the assessment of cost-effectiveness of immunizations. Often the costs of vaccination have been assessed by multiplying the total hourly costs of the regular working time of vaccinators (physician or nurse) with an educated guess, based on questionnaires, of the time used for administering a vaccine. In previous reports the costs of vaccination have ranged widely [7–10]. Previously the accurate time used for administering the vaccines of national vaccination program in a real-world setting has been measured in a T&M study conducted in six General Practitioner practices in the United Kingdom [9].

The aim of this study was to measure the time HCP used for different tasks during the CHC visits with a special emphasis on time used for administering vaccines.

## Methods

This T&M study was conducted in four CHCs in Finland during a three-month period (September to November 2017). We selected CHCs with at least 100 births per year. Two CHCs from eastern Finland and two from western Finland. From both sides of Finland we chose one bigger and one smaller town with rural areas. Furthermore, the CHC personnel interest in participation and availability of local study staff was required for the site selection. Data was gathered by two study nurses specially trained for the study conduct. After theoretical instruction they trained in the CHCs and observed a few visits prior to study start. Study focused on measuring the time required to perform pre-specified tasks as defined in Table 1. The pre-specified tasks were first gathered from the CHC statute [1] and then split to smaller tasks based on our experience on CHC work. The list of tasks was evaluated and approved by the CHC nurses before the observations began.

**Table 1:** Observed tasks for which times were measured.

Health care personnel observed		Task group
**Public health nurse PHN**	**Physician**	
PHN, total time	Physician, total time	Not included in any task group
**Actions**		
invitation to the room	invitation to the room	Documentation
measurements		Health check-up
physical status (skin, reflexes, senses etc)	physical status (skin, reflexes, senses etc)	Health check-up
development assessment	development assessment	Health check-up
**Discussions: Health counselling and discussions**		
breast feeding counselling	breast feeding counselling	Health counselling and discussions
Nutrition	nutrition	Health counselling and discussions
Sleeping	sleeping	Health counselling and discussions
day care	day care	Family matters
matters concerning parents	matters concerning parents	Family matters
matters concerning siblings	matters concerning siblings	Family matters
vaccination guidance	vaccination guidance	Vaccination
other matters	other matters	Health counselling and discussions
**Actions (vaccination)**		
vaccines from the fridge		Vaccination
preparing rota		Vaccination
preparing DTaP-IPV-Hib		Vaccination
preparing PCV		Vaccination
preparing MMR		Vaccination
preparing other vaccine(s)		Vaccination
administration rota		Vaccination
administration DTaP-Polio-Hib		Vaccination
administration PCV		Vaccination
administration MMR		Vaccination
administration other vaccine(s)		Vaccination
dispose of vaccination waste		Vaccination
vaccination documentation		Vaccination
**Monitoring after vaccination**		
monitoring after vaccination		Not included in any task group
**Actions**		
recording patient file, other (vaccination documentation excluded)	recording patient file, physician	Documentation
booking of next visit		Documentation
consultations, referrals, advice on medicinal care	consultations, referrals, prescriptions	Documentation

The observed visits were scheduled check-up visits during the first year of life. Details of the visits are presented in Fig 1. We aimed to observe 10 visits per each age group in each CHC to be able to see the possible differences in administering different kind of vaccines. As the visits available for observation was hard to predict it was allowed to observe more than 10 visits per age group per CHC to be able to get altogether around 40 observed visits per age group. All the visits observed after observation training visits were included in the study. In two age groups we were able to observe only round 20 visits: 2–4 weeks olds visits were observed if the visit took place in CHC and the 2 months visit was mainly a group visit in two CHCs and we did not observe those (Fig 1).

The whole process of a CHC visit involves a series of activities prior to the visit day (e.g. managing appointments to the visits, ordering vaccines and brochures, and cold-chain management), which should also be considered for a complete assessment of the resource use and cost implications of current CHC visits and vaccinations. The estimates of time used for these tasks were collected by interviewing selected PHNs who were responsible for the vaccine logistics in the CHC.

The visit could be chosen to be observed if at least one of the parents spoke fluent Finnish. Visits of children with congenital conditions or chronic diseases that required special attention were excluded from study. Observation of several consecutive visits of one child was allowed.

Trained observers recorded the time used for each pre-specified task using a stopwatch application (Ultimate Watch) on a tablet computer. The stopwatch application allowed the observers to measure time for more than one task at a time. Only one discussion and only one action stopwatch (see the table of tasks, Table 1) was allowed to be on simultaneously. The measurements began when the HCP invited the family in; at that moment the stopwatch measuring the total time HCP used for the visit (“nurse total” or “physician total”) and the “invitation to the room” stopwatch were started. The visit ended either when the family left the room or when the HCP finished the documentation tasks of the visit. The monitoring-after-vaccination stopwatch was started when the child got her/his first vaccine dose and was stopped when the family left the room. Thus, at most there could be four stopwatches on at the same time (PHN total, one action, one discussion, and monitoring after vaccination).

Time used for vaccination was separated to observed and non-observed time. The observed time included guidance, preparation and administering of vaccine, waste disposal and documentation. The non-observed time included vaccine logistics, taking care of cold chain, preparation of vaccination room and checking the patient file of the child. The time for preparing and administering of each vaccine dose was observed separately.

Labour costs were calculated by using the gross average labour costs of a public health nurse and physician (general practitioner) which were 20.80 and 49.70 Euros per hour, respectively [11]. Labour costs included social security contributions, holiday and sick leave pays.

Prices of consumables, presented in Table 2, were taken from publicly available sources [12].

**Table 2:** Costs of consumables including taxes from www.medkit.fi.

Consumable	Unit cost (€)
Kidneydish	0.12
Syringe	0.07
Needle	0.09
Plaster	0.03
Cellulose swab	0.01
Paper	0.02

The measured times were analysed in three groups: time used by PHN, time used by physician and the time PHN used at vaccination visits for different vaccination tasks. The vaccination visit was defined as a visit, where child got at least one vaccine dose. For each task and task group the used times are presented as means with 95% confidential limits (Cl) and range.

To describe the differences in the mean times used for each task in different CHCs (i.e. sites), we present the site range for task (or group of tasks). For comparison of observed time used for vaccination in different CHCs we present the mean values in each CHC with 95% CI. If the Cls did not overlap, the differences between the sites were considered statistically significant.

The Mann-Whitney U Test for independent -samples was used for analysis of the significance of the difference of the times of preparing and administering vaccines. Statistical analysis was made with IPM SPSS Statistics 27.

The study was approved by the Institutional Review Board of the Finnish Institute for Health and Welfare. For the T&M data collection the HCPs gave a written consent for the observations before the study began in the CHC and the families gave oral consent before the visit they attended. We did not gather personal data of the children or the personnel.

## Results

**Fig 2:**
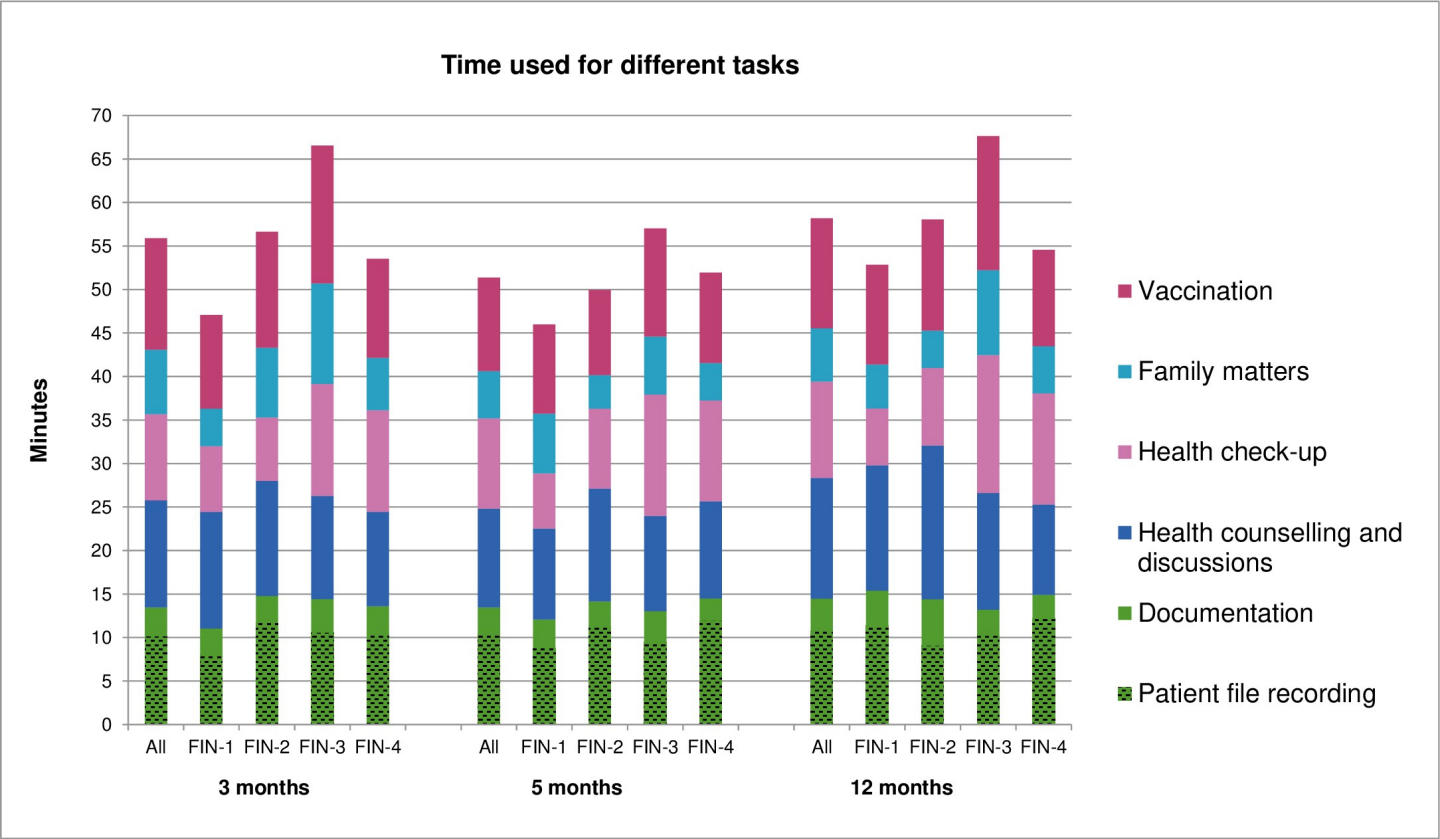
The observed time used for different task during 3, 5 and 12 month visits. The tasks belonging to each task group are shown in Table 1. Note: recording patient file is included in the documentation task group.

Altogether 325 CHC visits of under 13-month-old children conducted by 23 public health nurses were observed. The time reserved for visits was 20 to 60 minutes. On average, one visit took 49 (range 12–101) minutes of nurse’s time (Table 3). The average labour costs of a visit were 17 Euros. Among those 325 visits there were 122 visits including physician’s visits. We observed visits of 16 different physicians. The time reserved for physician was 20 minutes, but on average 23 (range 13–44) minutes were used. The labour costs of physicians were 19 (range 11–36) Euros per visit. Visits included different tasks which could be divided into five main groups: health check-up, health counselling and discussions, family matters, vaccination and documentation (Table 1). The mean time used for task groups varied slightly between the CHCs observed (Fig 2).

**Table 3:** Time used and cost of the visits observed. Time used for vaccinations during all visits.

	Visit											
	2–4 wk	4–6 wk		2 mo	3 mo	4 mo		5 mo	6 mo	8 mo		12 mo
**N of visits observed**	19^a^	42		21^b^	41	39		42	39	41		41
Range of N in CHCs	0–10	10–12		0–10	10–11	9–10		10–11	8–11	9–11		10–11
**Person performing visit**	PHN	PHN	Physician	PHN	PHN	PHN	Physician	PHN	PHN	PHN	Physician	PHN
Time reserved, mean	48	20	20	55	59	20	20	58	52	20	20	59
Time reserved, range	30–60			30–60	45–60			45–60	30–60			45–60
Time consumed, mean	44	18	23	52	58	16	22	52	46	19	24	60
Time consumed, range	19–70	8–32	14–44	30–70	34–101	7–29	13–41	31–68	25–82	8–29	13–39	46–79
Labour cost of visit, mean	15	6	19	18	20	6	18	18	16	7	20	21
Labour cost of visit, range	7–25	3–11	12–36	11–25	12–35	2–10	11–34	11–24	9–29	3–10	11–32	16–28
Sum of measured times of tasks^c^, mean	45	15	21	52	56	13	20	51	45	14	23	58
Sum of measured times of tasks, range	19–66	6–32	11–49	31–70	34–97	2–24	11–39	30–74	25–69	2–32	10–39	44–80
**Vaccinations** ^d^												
time used for vaccination, mean	1.23	2.78	0.02	7.64	12.79	0.39	0.003	10.76	3.38	1.09	0.04	12.64
time used for vaccination, median	0.68	1.88	0	7.18	12.15	0.17	0	11.14	2.5	0.18	0	12.18
time used for vaccination, range	0–5.2	0–9.7	0–0.8	2.7–15.1	6.6–24.6	0–2.7	0–0.1	0–20.4	0–11.7	0–4.6	0–1.5	1.0–23.9
**Visits with at least vaccine guidance**	16 (84%)	34 (81%)	1 (2%)	21 (100%)	41 (100%)	21 (54%)	1 (3%)	41 (98%)	36 (92%)	25 (59%)	1 (2%)	41 (100%)
**Visits with vaccines administrated**	1	20		18	40	0		39	16	10		39
doses given, mean	1	1		1	3.0	-		2.8	1.1	1		2.9
doses given, range	1	1		1	2–3	-		1–3	1–2	1		1–4

HN = public health nurse.

^a^Usually home visit, gathered if visit took place in CHC, unable to reach 10 visits from each CHC during the time study was conducted.

^b^Group visit in two CHCs, observations only from individual visits.

^c^Different tasks defined in Table 1.

^d^Vaccination includes: guidance, preparing, administering and documentation of vaccination.

**Fig 3:**
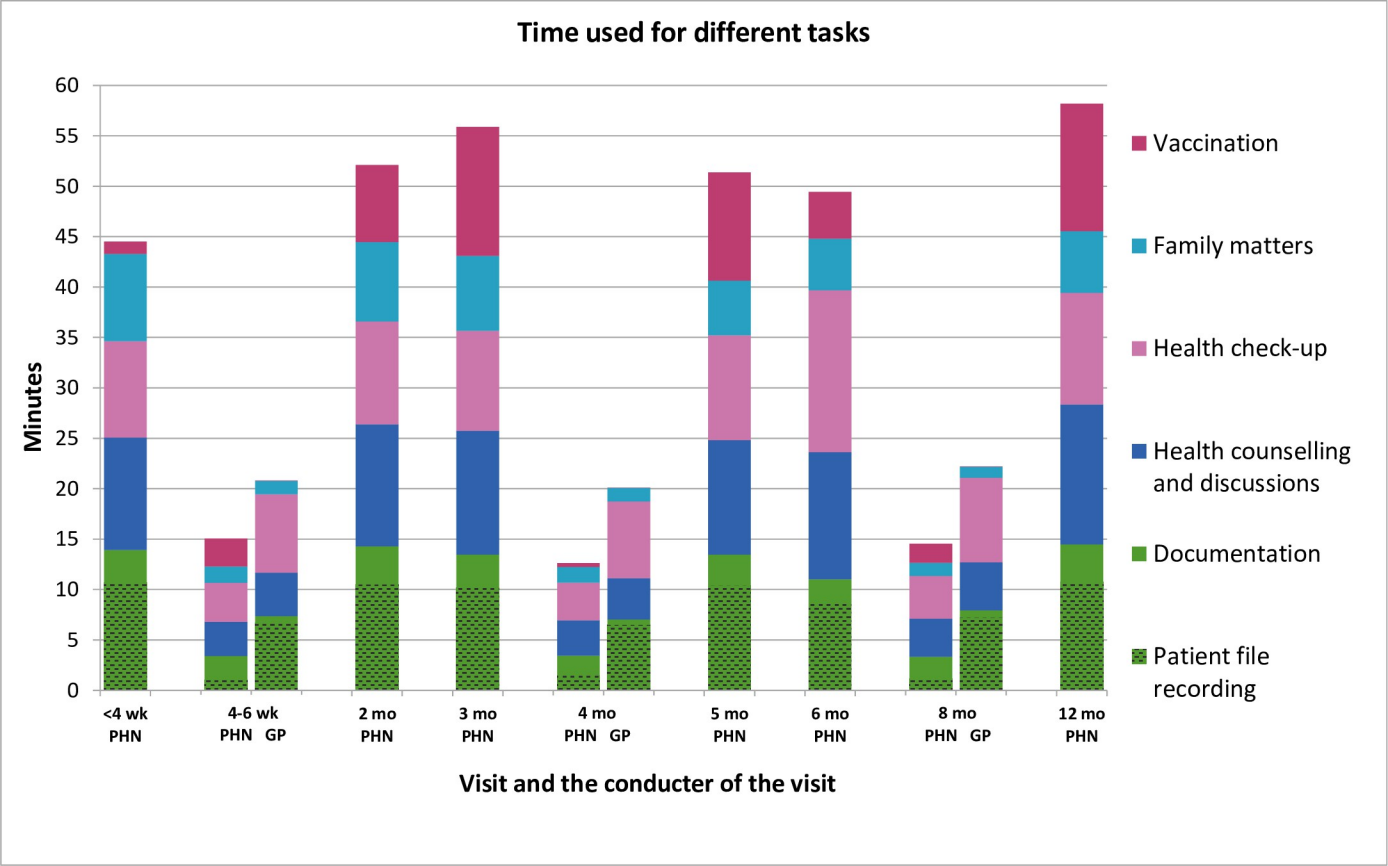
The time public health nurses (PHN) and physicians (GP) used for different task during the child’s visit to child-health-clinic. The tasks belonging to each task group are shown in Table 1. Note: recording patient file is included in the documentation task group.

Documentation tasks took a lot of time, about one quarter of the overall visit time (Fig 3). Furthermore, in the interview the PHNs reported that some of the documentation tasks, most often the patient file recording, cannot be done within the time reserved for the visit, but it’s left to be done later on. The second most time-consuming task group was health counselling and discussions about the matters concerning, for example, nutrition and sleeping (Fig 3).

Vaccines were administered at 183 visits. Most of the vaccines were given by the recommended schedule. Only a few vaccines were not given either because the child was ill or the parent(s) declined or postponed the administration of certain vaccines. Some doses were postponed due to the PHN’s recommendation, as some PHNs did not want to give four injections at one visit which would have been needed during influenza vaccination season at the 12-month visit (Fig 1).

Vaccination guidance was given additionally at 94 visits during which vaccinations were not given. Thus, some time was used for vaccination topic at least at 277 visits, which was 85% of all visits. Of the 48 visits during which vaccination topics were not addressed, 41 were physician visits. Actually, vaccine guidance was given by a physician only during 3 of the 122 observed physician visits (Table 3).

During the vaccination visits children got on average 2.4 (range 1–4) vaccine doses. The mean time used for vaccinations was 10.2 (range: 1.6–25, site range 9–12) minutes (Table 4). The time included guidance, preparation and administration of vaccine(s), waste disposal, and entering vaccination(s) to patient file, but not vaccine logistics or administrative tasks. Most vaccine doses in Finland were given at the age of 2, 3, 5 and 12 months, but some vaccines were given during other visits, especially during influenza vaccination season (end of October to January) (Table 3). During the 3, 5 and 12 months visits, which include 3 vaccine doses, the vaccinations took 11 to 13 minutes (Fig 2).

**Table 4:** The time and cost used for vaccination (A) and the effect of adding a vaccination dose to visit (B).

A								B		
Doses given	Visits	Time used to vaccination (min)			Costs of vaccination (€)			Effect of adding a dose		
		Mean	95% CI for mean		Mean	95% CI for mean			Increase in mean	
**1**	68	6.0	5.3	6.7	2.10	1.86	2.33	**Addition**	**time (min)**	**costs (€)**
**2**	6	10.5	8.1	13.0	3.69	2.84	4.53	**1->2**	4.5	1.59
**3**	106	12.8	12.1	13.5	4.49	4.24	4.73	**2->3**	2.3	0.80
**4**	3	14.5	10.6	18.3	5.06	3.72	6.41	**3->4**	1.6	0.57
**Any**	183	10.2	9.6	10.9	3.58	3.34	3.82	**average**	2.8	0.99

The average labour costs of vaccine administration were 3.58 (range 0.57–8.62) Euros per visit. Adding one vaccination dose for a visit increased the time spent on vaccination on average 2.8 minutes; and costs 0.99 Euros (Table 4).

**Fig 4:**
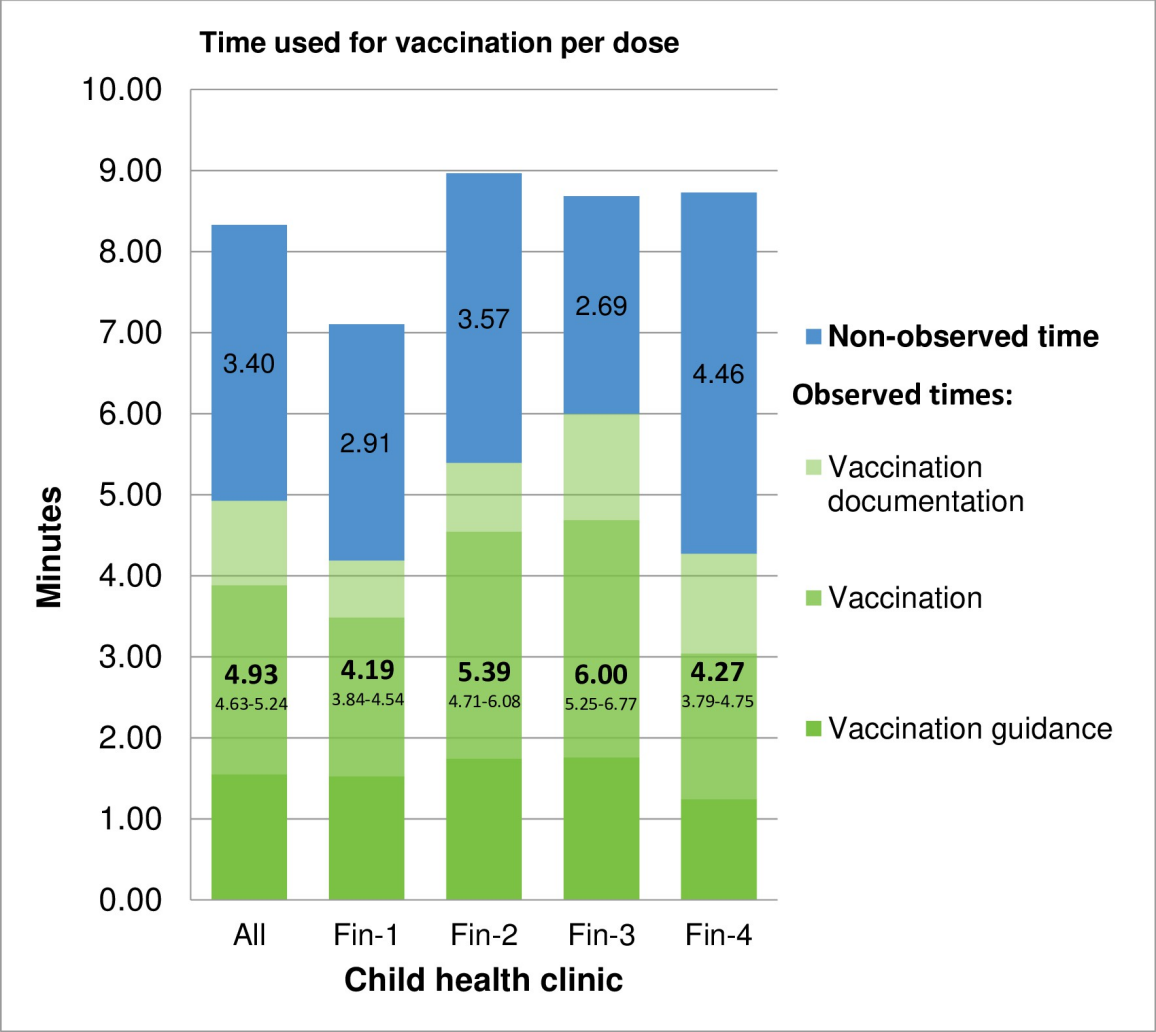
The observed and non-observed time used for one vaccination dose in different child health clinics.

**Fig 5:**
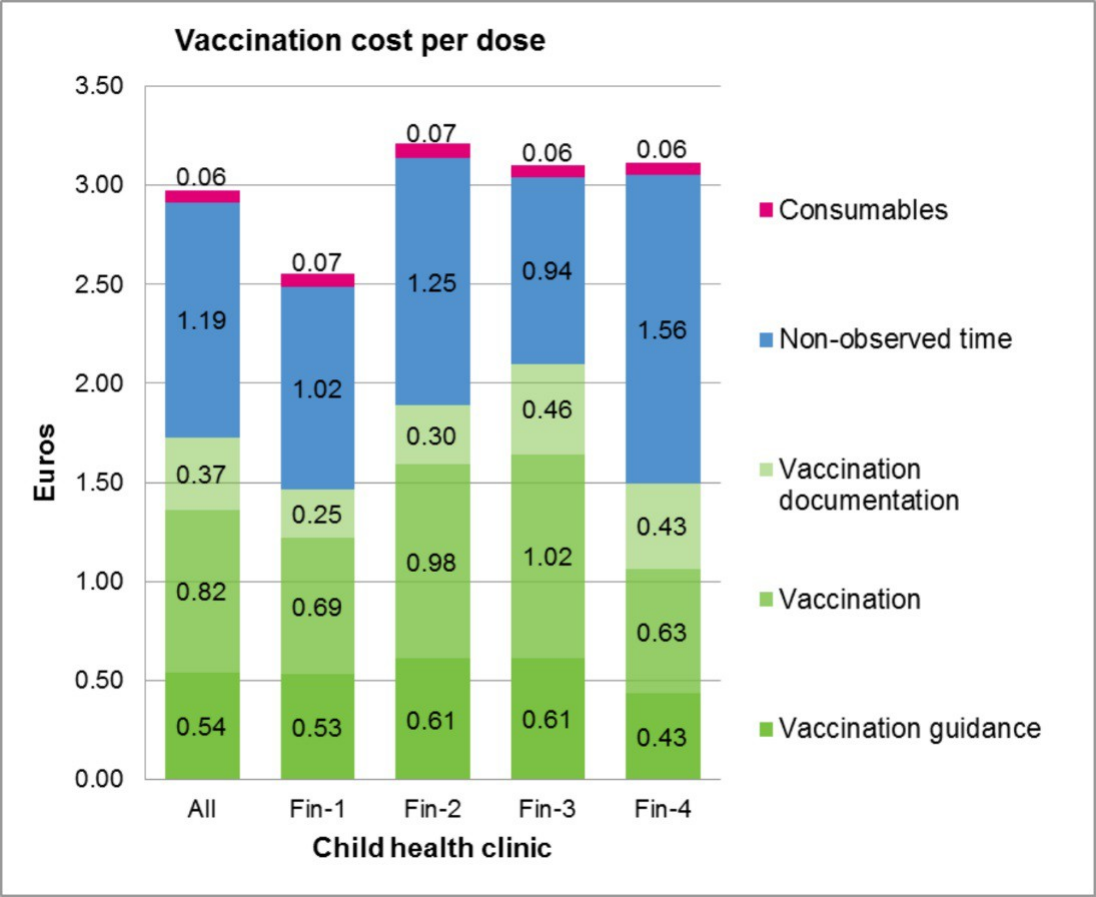
The total costs of giving one vaccine dose in Finnish CHC.

The mean observed time used for administering one vaccine dose was 4.9 (range 4.2–5.2; site range 4.2–6.0) minutes per dose. Nurses in site Fin-1 used significantly less time for vaccination when compared to nurses in sites Fin-2 and Fin-3 (Fig 4). No obvious reason for this difference could be found, but the difference was seen both in vaccination (which includes preparation and administration of vaccine(s) and waste disposal) and documentation. However, vaccination guidance took as much time and cost in every site (Fig 5). The non-observed time was on average 3.4 (site range 2.7–4.5) minutes per dose (Fig 4). Thus, the overall time used for vaccination was 8.3 (site range 7.1–9.0) minutes per dose. The observed labour costs of giving one vaccine dose were 1.73 Euros, and the total costs were 2.98 Euros including non-observed labour costs and consumables (Fig 5).

The time used for vaccination depended on the type of vaccine. The preparation and administration of oral vaccines (i.e. rota vaccine) took more time than giving injections. The mean time used for giving oral vaccine was 2.15 minutes (95%CL 1.94–2.36, range 0.4–10.2) and for injected vaccines the time used was 1.34 (95%CL 1.21–1.36, range 0.21–1.75) minutes (Table 5). Of the injected vaccines, the fully liquid vaccines (pneumococcal conjugate, hepatitis B and seasonal influenza vaccines) were quicker to prepare when compared to vaccines that needed reconstitution (diphteria-tetanus-pertussis-polio-*Haemophilus influenzae type B* and measles-mumps-rubella -vaccines) (Table 5).

**Table 5:** Observed time in minutes (A) and labour costs in Euros (B) used for preparing and administering different vaccines.

**A**		**Preparing (min)**					**Administering (min)**					**Total**				
	**Doses**	**Mean**	**95% CLs**			p	**Mean**	**95% CLs**			p	**Mean**	**95% CLs**			p
**Rota, oral**	115	0.57	0.51	0.64			1.58	1.38	1.77			2.15	1.94	2.36		
						<0.001					<0.001					<0.001
**Injected vaccines**	295	0.84	0.78	0.90			0.45	0.41	0.49			1.34	1.26	1.41		
**Reconstitution needed**	150	1.30	1.22	1.39			0.49	0.45	0.52			1.79	1.70	1.88		
*DTaP-IPV-Hib*	*116*	*1*.*34*	*1*.*24*	*1*.*44*		0.001	*0*.*48*	*0*.*43*	*0*.*52*		0.002	*1*.*81*	*1*.*71*	*1*.*92*		<0.001
*MMR*	*34*	*1*.*19*	*1*.*05*	*1*.*34*			*0*.*51*	*0*.*42*	*0*.*60*			*1*.*70*	*1*.*52*	*1*.*87*		
**fully liquid**	145	0.45	0.41	0.49			0.41	0.37	0.45			0.87	0.81	0.93		
*PCV*	*108*	*0*.*51*	*0*.*46*	*0*.*56*			*0*.*41*	*0*.*38*	*0*.*45*			*0*.*92*	*0*.*86*	*0*.*98*		
*Other*	*37*	*0*.*29*	*0*.*24*	*0*.*33*			*0*.*41*	*0*.*30*	*0*.*53*			*0*.*70*	*0*.*56*	*0*.*85*		
**B**		**Preparing (€)**					**Administering (€)**					**Total**				
	**Doses**	**Mean**	**95% CLs**			p	**Mean**	**95% CLs**			p	**Mean**	**95% CLs**			p
**Rota, oral**	115	0.20	0.18	0.22			0.55	0.48	0.62			0.75	0.68	0.82		
						<0.001					<0.001					<0.001
**Injected vaccines**	295	0.29	0.27	0.32			0.16	0.14	0.17			0.47	0.44	0.49		
**Reconstitution needed**	150	0.46	0.43	0.49			0.17	0.16	0.18			0.63	0.60	0.66		
*DTaP-IPV-Hib*	*116*	*0*.*47*	*0*.*43*	*0*.*50*		0.001	*0*.*17*	*0*.*15*	*0*.*18*		0.002	*0*.*63*	*0*.*60*	*0*.*67*		<0.001
*MMR*	*34*	*0*.*42*	*0*.*37*	*0*.*47*			*0*.*18*	*0*.*15*	*0*.*21*			*0*.*59*	*0*.*53*	*0*.*66*		
**fully liquid**	145	0.16	0.14	0.17			0.14	0.13	0.16			0.30	0.28	0.33		
*PCV*	*108*	*0*.*18*	*0*.*16*	*0*.*20*			*0*.*14*	*0*.*13*	*0*.*16*			*0*.*32*	*0*.*30*	*0*.*34*		
*Other*	*37*	*0*.*10*	*0*.*08*	*0*.*12*			*0*.*14*	*0*.*10*	*0*.*19*			*0*.*25*	*0*.*19*	*0*.*30*		

Other = 35 influenza and 2 hepatitis B vaccines.

The children vaccinated were monitored after vaccination on average 14.7 (range 13.7–15.7) minutes.

## Discussion

In this unique study we measured the time used for different tasks included in the scheduled CHC visits in Finland. Using the observed times and the average salary of health care personnel we were able to calculate the labour costs for the different tasks. The observed labour costs of a nurse were 17 Euros for the whole visit lasting on average one hour. The physician used for all the tasks on average 23 minutes per visit and the mean labour costs were 19 Euros. During the visits the growth and physical and psychosocial development of the child were checked, the mental health and psychosocial well-being of the family was assessed and health counselling was given to the family. Furthermore, the paediatric vaccines were administered according to the National Vaccination Programme. Administering one vaccine dose cost 3 Euros including observed and non-observed time. When only the observed time used for the vaccinations is included in the evaluation, adding one vaccine dose to a visit without other vaccinations would add costs two Euros and if a dose would be added to a visit with other vaccines the costs would be about one Euro.

In the Finnish CHCs the physicians are in charge of the vaccinations, but in practice PHNs take care of the vaccines and administer them. In this study, physicians gave vaccination guidance at only 3 out of 122 physician visits. If physicians would have given the vaccines the costs of vaccination would have been more expensive because of the considerably higher labour costs.

This study was the first one to measure the times used for different tasks at the Finnish CHC visits. With the T&M method the actual costs of the various parts of the visit could be estimated. This study revealed that HCP use the time reserved for the predefined tasks. Documentation of the visit was time-consuming as reported also in a T&M study evaluating the time and costs of different number of vaccinations given to child [4].

The costs of administering the vaccines of the National Vaccination Programme have been previously evaluated in United Kingdom (UK) by the T&M method [9] and with activity logs [13]. The observed times used by nurses for vaccinations were quite similar, about 10 minutes, in Finland and the UK, even though the systems are different. In Finland, children visit CHCs 9 times during the first year of life and the visits include many other topics in addition to vaccination. In the UK the vaccination is the actual reason for visiting the clinic.

The whole process of CHC visits involves also a series of activities prior to the visit day (e.g. reserving the visit, ordering vaccines and brochures, cold-chain management), which were also considered for a complete assessment of the resource use and cost implications of the CHC visits. Unfortunately, we were not able to observe and measure the time used for those tasks, thus the time and costs of those were evaluated by interviews. The time used for the vaccine logistics varied in the CHCs because some clinics were responsible for all the vaccines given in the health station (including vaccines to adults) and others took care of the vaccines they needed themselves at CHC.

The CHC clinic visits in Finland have not been previously studied by T&M methods, but the time and costs of vaccinations have been assessed for the use of cost-effectiveness studies [14] by using total hourly cost of regular working time of a nurse [11]. Previously, 10 minutes per dose have been used as the time used for vaccination in Finnish cost-benefits calculations [14]. A T&M study on paediatric vaccination in the UK reported mean health care professional time for a vaccination process of 19.6 minutes, of which 9.5 minutes during the vaccination day (T&M data) and 10.1 minutes prior to the vaccination day (based on interviews) [9]. Other literature estimates for a single vaccine dose administration ranged between 17.3 minutes in the United States (US) (activities prior to and on the vaccination day) [7] and 23.8 minutes in New Zealand (vaccination day only) [8] based on the use of time diaries and questionnaires, respectively. Both studies suggested that their main limitation was the small sample size. In the US study it was also identified that self-reported, even though diary data, time estimates were not as accurate as observed times would have been [7]. In New Zealand there was a big variability in the results among practices [8]. We had four CHCs in the study and the number of observations was quite high. We got quite uniform results, because the subject matters of the visits have been regulated in a statute, leading to similar practises in the CHCs.

We found two T&M studies made in New York, USA. In both studies observations were done in 7 clinics. In a study where 164 vaccination visits of children were observed, the median time the HCP used for a visit was 21.4 minutes. In that study most children got three vaccinations and the time included only the observed time [15]. In another T&M study 102 seasonal influenza vaccination visits were observed. The median observed time used for the visit was 14 minutes [16].

The time estimates from the afore-mentioned studies are not easily comparable due to different definitions and methods used. Also, the fact that vaccination is a part of routine health check-up visits makes the Finnish system different from other studies [7–9, 13, 15, 16].

The children were monitored after vaccination on average 15 minutes after the first vaccine dose. The PHNs work was organised so that they were able to monitor children the recommended time [17, 18] which is not the case when the whole vaccination visit lasts 20 minutes or less [9, 13, 15, 16].

The type of vaccine played an important role in the time used for vaccination. In previously published studies it has been noted that it is quicker to give one shot instead of three shots [4], and slower to give a vaccine needing reconstitution when compared to ready-to-inject vaccine [5, 19]. Both results were observed also in our study. Furthermore, we showed that administering an oral vaccine is slower than giving an injection.

In this study we were able to observe the PHNs and physicians in four CHCs. It could be claimed that more CHCs would have given better generalizability to the results. As the CHC visits are regulated by statute [1], we think that the practices in different CHC are very similar as observed in our study.

We chose to collect the time used for vaccine logistics and other administrative tasks by interviews, which led to a wide site range of the time used for these non-observed tasks. The time of logistics would probably have varied widely also if observed directly, as the logistics arrangements were very different in different CHC as explained above.

We excluded visits of children whose parent(s) did not speak Finnish and visits of children with congenital or chronic diseases to get an overview of the routine work. If the excluded visits had been observed probably the overall time would have been longer and the time used for some tasks might have varied more. These excluded visits were rare in the CHCs observed and thus we do not think that they have an impact on the generalizability of the results.

To be able to understand the differences in the observed times of vaccination we should have observed also the environment of the visit rooms and the facilities. For example, the location of the fridge where the vaccines were (there was not fridge in every appointment room) might have lengthen the time used for vaccination. Unfortunately, we did not record this kind of details accurately enough.

## Conclusions

Administering of one vaccine dose took about eight minutes and cost three Euros in the Finnish CHCs. Administering of the vaccines of the Finnish vaccination programme is relatively simple and inexpensive because Finnish children have regular scheduled visits to CHCs.

## Supporting information

S1 Dataset Minimum dataset with metadata. (XLSX)
